# Recurrence After Colectomy for Locally Advanced Colon Cancer: Experience from a Developing Country

**DOI:** 10.1007/s13193-022-01672-x

**Published:** 2022-11-01

**Authors:** Artur M. Sahakyan, Andranik Aleksanyan, Hovhannes Batikyan, Hmayak Petrosyan, Shushan Yesayan, Mushegh A. Sahakyan

**Affiliations:** 1 Department of Surgery N1, Yerevan State Medical University After M. Heratsi, Yerevan, Armenia; 2 Department of General and Abdominal Surgery, ArtMed MRC, Yerevan, Armenia; 3 Clinic of Surgery, Mikaelyan Institute of Surgery, Yerevan, Armenia; 4 Department of Anesthesiology, ArtMed MRC, Yerevan, Armenia; 5 The Intervention Center, Oslo University Hospital Rikshospitalet, Sognsvannsveien 20, 0424 Oslo, Norway; 6 Department of Research & Development, Division of Emergencies and Critical Care, Oslo University Hospital, Oslo, Norway

**Keywords:** Colon, Adenocarcinoma, Colectomy, Recurrence

## Abstract

Risk factors for disease recurrence following curative resection for locally advanced colon cancer (LACC) remain unclear as conflicting results have been reported in the literature. The aim of this study was to examine these factors in the setting of developing country’s health care system affected by limited accessibility to the multimodal cancer treatment. Patients who had undergone curative colon resection for LACC between 2004 and 2018 were included. Data were obtained from a prospectively maintained database. Factors associated with disease recurrence, types of recurrence and recurrence-free survival were studied. A total of 118 patients with LACC were operated within the study period. Median follow-up was 36 (2-147) months. Adjuvant therapy was used in 41 (34.7%) patients and 62 (52.5%) were diagnosed with recurrence. In the multivariable analysis, disease recurrence was associated with tumor and nodal stages, as well as with the lymph node yield. Local recurrence, distant metastases, and peritoneal carcinomatosis were observed in 8 (6.8%), 30 (25.4%), and 24 (20.3%) patients, respectively. Early recurrence was diagnosed in 27 (22.9%) cases with peritoneal carcinomatosis being its most common type. Preoperative serum CA 19–9 levels, tumor, and nodal stages were linked to recurrence-free survival in the univariable analysis. Only tumor stage remained such in the multivariable model. Our findings suggest that lymph node yield, tumor, and nodal stages are associated with recurrence following curative resection for LACC.

**Supplementary Information:**

The online version contains supplementary material available at 10.1007/s13193-022-01672-x.

## Introduction

Colon cancer is listed among the most common cancers worldwide representing one of the leading causes of cancer-related death [1, 2]. While multimodal treatment based on surgical resection remains the only curative option in these patients, disease recurrence occurs in 30–40% of cases [3]. Its risk is especially high within the first 6–12 months of surgery [4, 5]. Prognostic factors such as colon lumen obstruction, tumor location, disease stage, lymph node metastases, tumor grade, and perineural and vascular invasion are shown to be associated with recurrence [5, 6].

Approximately 10–15% of patients with colon cancer are diagnosed with locally advanced disease [7]. Despite curative resection, 35–40% of these patients experience recurrence [8–10]. Some authors observed an increased risk of locoregional recurrence following surgery in locally advanced colon cancer (LACC) [9], while others reported high incidence of distant metastases and peritoneal carcinomatosis [10, 11]. At the same time, most of these reports come from the centers or countries with high accessibility to multimodal treatment and chemotherapy specifically. In contrast, there is a limited literature from developing countries affected by the lack of resources in the healthcare system.

Assessment of the risk factors for recurrence in LACC requires scrutiny considering the choice of adequate treatment strategy, especially in the setting of possible limitations for multimodal treatment. The aim of this study was to examine recurrence and associated outcomes following resection for LACC based on the experience from a developing country.

## Methods

### Study Design and Patients

Patients with LACC who had undergone colon resection between 2004 and 2018 were included in this study. Data were retrospectively obtained from a prospectively maintained database, which includes information on patient demographics, clinical characteristics, perioperative results, pathology findings, and long-term outcomes. The impact of these parameters on disease recurrence, types of recurrence, and recurrence-free survival was studied. Patients diagnosed with colon cancer originating from a histological entity other than adenocarcinoma were excluded from the analysis. So were those diagnosed with the distant metastases (M1a, M1b, M1c) at the time of surgery.

Patient management and follow-up have been described elsewhere. None of the patients had received neoadjuvant therapy and all of those were submitted to open surgery. Clinical examination, chest X-ray, ultrasound of the abdomen, and measurement of serum cancer markers were performed 3 and 6 months after surgery and repeated every 6 months throughout the first 5 years. Chest and abdominal computed tomography was done 1 year after surgery and repeated annually.

### Definitions

LACC was defined as stage pT4a and pT4b colon cancer confirmed by the pathology report. Complications following surgery were defined and classified according to Clavien and Dindo [12]. Grade ≥ II complications were registered in the database. Tumor size was defined as its largest dimension measured microscopically by the pathologist.

Recurrence was defined as radiological evidence of a soft tissue around surgical site or distant metastases/carcinomatosis. Tumor recurrence was classified as local, distant metastasis and peritoneal carcinomatosis. Early recurrence was defined as disease relapse within the first year of surgery as suggested elsewhere [13, 14]. Patients without recurrence were censored at the last follow-up. Recurrence-free survival was estimated from the date of surgery until the date of recurrence diagnosis or the date of censoring. Follow-up data was available in all patients.

### Statistics

Data were presented as either categorical or continuous. The latter was expressed in means (± standard deviation) or medians (range) depending on data distribution. Categorical data was presented in numbers (percentages).

The two-sample *t*-test was used to compare means, while the Mann–Whitney *U* test was used for medians. The chi-square test or Fisher’s exact test was applied to compare the categorical data. *P-*value < 0.05 was considered statistically significant. Parameters that were significant (*p* < 0.05) in the univariable analysis were added to the multivariable model. Binary logistic regression model was used when the outcome parameter was binary. Cox regression model was applied for recurrence-free survival analysis.

## Results

### Recurrence

Median follow-up was 36 (2–147) months. Of 118 patients eligible for the analysis, 62 (52.5%) were diagnosed with disease recurrence, while 56 (47.5%) were recurrence free at the last follow-up. Demographics and perioperative data were similar between the patients with and without recurrence (Table 1). The incidence of pT4b was significantly higher among those who had recurrence (82.3 vs 42.9%, *p* = 0.001). So were the proportion of multi-visceral resections, presence of < 12 lymph nodes in the specimen, and the incidence of lymph node–positive disease. Mean lymph node yield was greater among those who did not experience recurrence (18 vs 13, *p* =0.018) The proportion of patients who had received adjuvant chemotherapy was lower among those with recurrence (25.8 vs 44.6%, *p* = 0.032). Parameters associated with disease recurrence in the univariable analysis were added to the multivariable model (Table 2). Higher tumor and nodal stages remained as independent predictors for disease recurrence, while the increasing number of retrieved lymph nodes negatively correlated with the recurrence. Adjuvant chemotherapy and multi-visceral resections were not independent predictors for disease recurrence in the multivariable analysis.

**Table 1:** Demographics and clinical characteristics among the patients with and without recurrence following colon resection for locally advanced colon cancer

Parameters	Recurrence (*n* = 62)	No recurrence (*n* = 56)	*p*-value
Age, years, mean (± SD)	61.1 (13.1)	63.8 (9.7)	0.26
Body mass index, kg/m^2^, mean (± SD)	27.8 (5.1)	26.5 (4.5)	0.21
Gender (male), *n* (%)	34 (54.8%)	35 (62.5%)	0.4
Presence of comorbidities, *n* (%)	56 (90.3%)	50 (89.3%)	0.85
Tumor location in the colon, *n* (%)			0.86
Right	22 (35.5%)	19 (33.9%)	
Left/transverse	40 (64.5%)	37 (66.1%)	
Preoperative CEA, ng/ml, mean (± SD)	16.7 (4.7)	15.9 (3.4)	0.93
Preoperative CA 19–9, U/ml, mean (± SD)	29 (6.2)	66 (21.9)	0.35
Red blood cell transfusion, *n* (%)	6 (9.7%)	11 (19.6%)	0.12
Morbidity (≥ II C–D), *n* (%)	6 (9.7%)	6 (10.7%)	0.85
Anastomosis leakage, *n* (%)	4 (6.5%)	0 (0%)	0.12
Relaparotomy, *n* (%)	2 (3.2%)	2 (3.6%)	1.0
pT4b stage, *n* (%)	51 (82.3%)	24 (42.9%)	0.001
Multi-visceral resection, *n* (%)	37 (59.7%)	20 (35.7%)	0.009
Tumor size ≥ 6 cm, *n* (%)	50 (80.6%)	52 (92.9%)	0.053
pN stage, *n* (%)			0.021
N0	24 (38.7%)	36 (64.3%)	
N1	20 (32.3%)	10 (17.9%)	
N2	18 (29%)	10 (17.9%)	
Detected lymph nodes, mean (± SD)	13 (7)	18 (12)	0.018
≥ 12 detected lymph nodes	24 (38.7%)	39 (69.6%)	0.001
Positive lymph nodes, mean (± SD)	4 (3)	6 (6)	0.37
Lymph node ratio, mean (± SD)	0.34 (0.24)	0.33 (0.29)	0.92
Low tumor grade, *n* (%)	8 (12.9%)	10 (17.9%)	0.87
R1 resection margin^a^	2	1	0.56
Adjuvant chemotherapy, *n* (%)	16 (25.8%)	25 (44.6%)	0.032

^a^Incomplete data

**Table 2:** Multivariable analysis of risk factors for recurrence after colectomy locally advanced colon cancer

Parameters	Odds ratio (95% CI)	*p*-value
T4b stage (vs. T4a)	7.89 (1.66–37.54)	0.009
Multi-visceral resection (vs. standard)	0.39 (0.08–1.88)	0.24
Detected lymph nodes	0.9 (0.85–0.97)	0.003
pN stage (vs. pN0)		
pN1	6.36 (1.61–25.1)	0.008
pN2	4.53 (1.23–16.59)	0.022
Adjuvant chemotherapy	0.47 (0.23–1.96)	0.24

Uni- and multivariable analyses were performed to identify prognostic factors for recurrence-free survival (Table 3). Preoperative serum CA 19–9 levels, and tumor and nodal stages were significant in the univariable analysis. However, only tumor stage remained such in the multivariable model.

**Table 3:** Prognostic factors for recurrence-free survival following colon resection for locally advanced colon cancer

Variable	Univariable		Multivariable	
	HR (95% CI)	*p*-value	HR (95% CI)	*p*-value
Age, years	0.98 (0.95–1.01)	0.27		
Obesity (vs. normal weight)				
Overweight	1.84 (0.76–4.48)	0.18		
Obese	2.26 (0.84–6.08)	0.11		
Male gender (vs. female)	1.15 (0.64–2.07)	0.65		
Comorbidities	1.01 (0.91–1.11)	0.94		
Colon obstruction	0.73 (0.32–1.67)	0.45		
CEA > 3 ng/ml	0.85 (0.43–1.71)	0.65		
CA 19–9 > 37 U/ml	2.62 (1.29–5.33)	0.008	1.77 (0.81–3.85)	0.15
Right (vs. left/transverse colon)	1.07 (0.58–1.96)	0.83		
≥ 2 organ resections	1.38 (0.61–3.09)	0.44		
Red blood cell transfusion	0.83 (0.35–1.97)	0.67		
Postoperative complications	1.47 (0.62–3.52)	0.38		
pT4b stage (vs. T4a)	3.69 (1.84–7.37)	0.001	2.74 (1.25–6.01)	0.012
Tumor size ≥ 6 cm	0.66 (0.31–1.43)	0.29		
Lymphatic invasion	0.66 (0.3–1.46)	0.31		
Low tumor grade	1.15 (0.48–2.77)	0.76		
pN stage (vs. pN0)				
pN1	2.34 (1.18–4.66)	0.016	2.02 (0.84–4.88)	0.12
pN2	2.65 (1.26–5.57)	0.01	2.12 (0.83–5.39)	0.12
Detected lymph nodes	0.97 (0.94–1.01)	0.17		
≥ 12 detected lymph nodes (vs. < 12)	0.62 (0.34–1.13)	0.12		
Lymph node ratio	1.27 (0.22–7.29)	0.79		
Adjuvant chemotherapy	0.72 (0.39–1.33)	0.29		

### Recurrence Types

Local recurrence was observed in 8 (6.8%) patients, while distant metastases and peritoneal carcinomatosis were observed in 30 (25.4%) and 24 (20.3%) cases, respectively. Specific characteristics of the patients diagnosed with the distant metastases or peritoneal carcinomatosis are shown in Supplementary Table 1. Multi-visceral resections were more frequent in patients eventually diagnosed with the distant metastases compared to those with peritoneal carcinomatosis and without recurrence (73.3 vs. 45.8 vs. 35.7%, *p* = 0.004, respectively). pT4b stage was more common among the patients with the distant metastases and peritoneal carcinomatosis (76.7 vs. 87.5 vs. 42.9%, *p* = 0.001). Patients without recurrence had a higher incidence of ≥ 12 lymph nodes in the specimen and lymph-node negative LACC compared to those with distant metastases and peritoneal carcinomatosis (64.3 vs. 40 vs. 33.3%, *p* = 0.015, respectively). The use of adjuvant chemotherapy was significantly lower in patients who eventually developed peritoneal carcinomatosis when compared to those without recurrence.

Early recurrence was diagnosed in 27 (22.9%) patients including 4 with local recurrence, 10 with distant metastases, and 13 with peritoneal carcinomatosis. Performing multi-visceral resection and pN0 was associated with the lower incidence of early recurrence (Supplementary Table 2). In contrast, pN2 and the greater number of positive lymph nodes positively correlated with the early recurrence.

## Discussion

In this study, recurrence rate following curative resection for LACC was 52.5%, which is higher than reported in the literature [9, 15, 16]. At the same time, the proportion of patients that had received adjuvant chemotherapy was only 34.7%. Although adjuvant chemotherapy was not a significant predictor for recurrence in the multivariable analysis, one should pay attention that its application was still significantly low when comparing with the literature data (41.7–72.8%) [8, 9, 15, 17–19]. There can be various reasons to relatively uncommon use of adjuvant chemotherapy in our patients. First and foremost, this should be attributed to the economy as costs for chemotherapy are normally not covered by the healthcare system or health insurance. Second, some patients rejected the recommendation for chemotherapy due to socio-psychological and cultural factors. In their population-based multicenter cohort study, Teufel and co-workers suggest significant benefits for adjuvant chemotherapy in terms of its impact on recurrence, recurrence-free survival, and overall survival in patients with T4N0M0 colon cancer [20]. Several studies suggest promising results also for neoadjuvant chemotherapy in patients with LACC [21, 22]. Since none of our patients had received neoadjuvant therapy, its potential benefits could not be tested on this cohort.

Tumor and nodal stages as well as lymph node yield were associated with recurrence following resection for LACC. Furthermore, higher tumor stage was a negative predictor for recurrence-free survival. Clinical significance of these parameters has already been reported in the literature [8, 11, 15, 18]. Interestingly, though, our findings on tumor stage somewhat contradict to those reported by Aoki and co-workers, which found that pT4a was a significant predictor for shorter recurrence-free survival compared with pT4b [23]. At the same time, more than half of their patients were operated on laparoscopically, while our patients underwent exclusively open surgery. This fact merits attention in the light of previously highlighted prognostic impact of surgical approach on disease recurrence [11, 15].

The rates for different types of recurrence observed in this study are in line with those reported in the recently published international, multicenter study [8]. Moreover, the association between pathology findings (pT and pN stages) and the development peritoneal carcinomatosis confirms previously published results [8, 15, 18]. As mentioned, some authors have reported heterogenous cohorts including both open and laparoscopic procedures thereby trying to address the role of surgical approach in recurrence pattern for LACC [11, 15, 17, 23]. In that context, our study may offer some benefits as the study group was homogenous in terms of surgical approach used, which allows to focus on other important parameters.

Greater number of positive lymph nodes was associated with early recurrence (within 1 year of surgery), while performing multi-visceral resections and pN0 negatively correlated with early recurrence. The effect of multi-visceral resections on early recurrence merits attention as one can argue that in the light of such findings, multi-visceral resections should be strongly considered in patients not willing to proceed with adjuvant chemotherapy. At the same time, statistical significance of this parameter for early recurrence could not be confirmed as multivariable analysis was not possible due to the small sample size. Moreover, multi-visceral resections were not associated with overall recurrence when adjusted for confounders. To the best of our knowledge, no studies reporting early recurrence following surgery for LACC have been published to date. In contrast, some studies addressed this issue without differentiating between colon and rectal cancer or different pT stages for colon cancer [13, 24].

There are several limitations worth mentioning. First and foremost, our findings might be affected by the retrospective design and inherent biases of this study. Second, the sample size was relatively small, which did not allow for thorough subgroup analysis, especially when considering risk factors for different types of disease recurrence. Third, data on pathology parameters such as perineural invasion, R-status, and lymphatic and vascular invasion were incomplete, so these were left out of the analysis.

In conclusion, lymph node yield, and tumor and nodal stages are independent predictors for disease recurrence after curative resection for LACC, while tumor stage is associated with recurrence-free survival.

## Supplementary Information

ESM 1 (DOCX 22.3 KB)

## Data Availability

The datasets used and/or analyzed during the current study are available from the corresponding author on reasonable request.
