# Supplementary material for: Recurrence After Colectomy for Locally Advanced Colon Cancer: Experience from a Developing Country
Source: Indian J Surg Oncol. 2022 Nov 1;14(2):339–44. doi: 10.1007/s13193-022-01672-x (PMC10267088; doi:10.1007/s13193-022-01672-x)
Supplement: Supplementary file 1 — (DOCX 22.3 KB) [file 13193_2022_1672_MOESM1_ESM.docx]

**Supplementary table 1. Patient and disease characteristics among those diagnosed with distant metastases and peritoneal carcinomatosis after colon resection for locally advanced colon cancer.**

| **Parameters** | **Distant metastases**  **(n=30)** | **Carcinomatosis**  **(n=24)** | **No recurrence**  **(n=56)** | **p-value** |
| --- | --- | --- | --- | --- |
| Age, years, mean (±SD) | 64.3 (9.8) | 56.1 (15.6) | 63.8 (9.7) | 0.057 |
| Body mass index, kg/m^2^, mean (±SD) | 27.5 (5.5) | 27.6 (5.2) | 26.5 (4.5) | 0.64 |
| Gender (male), n (%) | 19 (63.3%) | 12 (50%) | 35 (62.5%) | 0.53 |
| Tumor location in the colon, n (%) |  |  |  | 0.91 |
| Right | 10 (33.3%) | 7 (29.2%) | 19 (33.9%) |  |
| Left/Transverse | 20 (66.7%) | 17 (70.8%) | 37 (66.1%) |  |
| Preop CEA, ng/ml, mean (±SD) | 10.9 (1.6) | 7.9 (1.3) | 15.9 (3.4) | 0.6 |
| Preop CA 19-9, U/ml, mean (±SD) | 45.9 (9.3) | 18.6 (2.6) | 66.4 (21.9) | 0.66 |
| Red blood cell transfusion, n (%) | 4 (13.3%) | 2 (8.3%) | 11 (19.6%) | 0.44 |
| Multi-visceral resection, n (%) | 22 (73.3%) | 11 (45.8%) | 20 (35.7%) | 0.004^┼,^ * |
| ≥ 2 organ resection, n (%) | 5 (16.7%) | 2 (8.3%) | 6 (10.7%) | 0.68 |
| Morbidity (≥ II C-D), n (%) | 4 (13.3%) | 1 (4.2%) | 6 (10.7%) | 0.59 |
| Anastomosis leakage, n (%) | 2 (6.7%) | 1 (4.2%) | 0 (0%) | 0.12 |
| pT stage, n (%) |  |  |  | 0.001^┼, ╪^ |
| T4a | 7 (23.3%) | 3 (12.5%) | 32 (57.1%) |  |
| T4b | 23 (76.7%) | 21 (87.5%) | 24 (42.9%) |  |
| Tumor size ≥ 6cm, n (%) | 24 (80%) | 19 (79.2%) | 52 (92.9%) | 0.11 |
| pN stage, n (%) |  |  |  |  |
| N0 | 12 (40%) | 8 (33.3%) | 36 (64.3%) | 0.015^┼, ╪^ |
| N1 | 11 (36.7%) | 6 (25%) | 10 (17.9%) | 0.15 |
| N2 | 7 (23.3%) | 10 (41.7%) | 10 (17.9%) | 0.075 |
| Detected lymph nodes, mean (±SD) | 13 (6) | 13 (8) | 18 (12) | 0.09 |
| ≥ 12 detected lymph nodes | 12 (40%) | 9 (37.5%) | 39 (69.6%) | 0.005^┼, ╪^ |
| Positive lymph nodes, mean (±SD) | 5 (4) | 4 (3) | 6 (5) | 0.79 |
| Lymph node ratio, mean (±SD) | 0.44 (0.23) | 0.27 (0.25) | 0.33 (0.29) | 0.47 |
| Low tumor grade, n (%) | 4 (13.3%) | 3 (12.5%) | 10 (17.9%) | 1.0 |
| R1 resection margin, n (%) | 0 (0%) | 1 (4.2%) | 1 (1.7%) | 0.33 |
| Adjuvant chemotherapy, n (%) | 9 (30%) | 4 (16.7%) | 25 (44.6%) | 0.045 ^╪^ |

* p-value was statistically significant between the group with distant metastases and those with peritoneal carcinomatosis,

^┼^ p-value was statistically significant between the group with distant metastases and those without recurrence,

^╪^ p-value was statistically significant between the group with peritoneal carcinomatosis and those without recurrence.

**Supplementary table 2. Perioperative results and pathology data in patients diagnosed with early and late recurrence following colon resection for locally advanced colon cancer.**

| **Parameters** | **Early recurrence**  **(n=27)** | **Late recurrence**  **(n=35)** | **p-value** |
| --- | --- | --- | --- |
| Age, years, mean (±SD) | 58.4 (12.1) | 63.2 (13.8) | 0.28 |
| Body mass index, kg/m^2^, mean (±SD) | 27.8 (5.7) | 27.9 (4.6) | 0.93 |
| Gender (male), n (%) | 14 (51.9%) | 20 (57.1%) | 0.68 |
| Tumor location in the colon, n (%) |  |  | 0.39 |
| Right | 8 (29.6%) | 14 (40%) |  |
| Left/Transverse | 19 (70.4%) | 21 (60%) |  |
| Preoperative CEA, ng/ml, mean (±SD) | 33.6 (6.9) | 3.5 (3.8) | 0.08 |
| Preoperative CA 19-9, U/ml, mean (±SD) | 27.2 (4.6) | 30.5 (7.4) | 0.88 |
| Red blood cell transfusion, n (%) | 1 (3.7%) | 5 (14.3%) | 0.22 |
| Multi-visceral resection, n (%) | 12 (44.4%) | 25 (71.4%) | 0.032 |
| ≥ 2 organ resection, n (%) | 3 (11.1%) | 5 (14.3%) | 1.0 |
| Morbidity (≥ II C-D), n (%) | 2 (7.4%) | 4 (11.4%) | 0.69 |
| Anastomosis leakage, n (%) | 1 (3.7%) | 3 (8.6%) | 0.63 |
| pT stage, n (%) |  |  | 1.0 |
| T4a | 5 (18.5%) | 6 (17.1%) |  |
| T4b | 22 (81.5%) | 29 (82.9%) |  |
| Tumor size ≥ 6cm, n (%) | 24 (88.9%) | 26 (74.3%) | 0.15 |
| pN stage, n (%) |  |  |  |
| N0 | 6 (22.2%) | 18 (51.4%) | 0.019 |
| N1 | 7 (25.9%) | 13 (37.1%) | 0.35 |
| N2 | 14 (51.9%) | 4 (11.4%) | 0.001 |
| Detected lymph nodes, mean (±SD) | 14.4 (5.9) | 11.8 (7.6) | 0.27 |
| ≥ 12 detected lymph nodes | 14 (51.9%) | 10 (28.6%) | 0.062 |
| Positive lymph nodes, mean (±SD) | 5.8 (3.7) | 2.4 (1.5) | 0.023 |
| Lymph node ratio, mean (±SD) | 0.39 (0.24) | 0.22 (0.17) | 0.13 |
| Low tumor grade, n (%) | 3 (17.6%) | 5 (19.2%) | 1.0 |
| R1 resection margin, n (%) | 0 (0%) | 2 (10.5%) | 0.49 |
| Adjuvant chemotherapy, n (%) | 8 (29.6%) | 8 (22.9%) | 0.55 |
